# Peritoneal-cutaneous fistula successfully treated at home: A case report and literature review

**DOI:** 10.1515/med-2020-0245

**Published:** 2020-11-14

**Authors:** Mirosław Kiedrowski, Eliza Mokrosz

**Affiliations:** Ewdomed Ewa Szulecka Foundation and Home Hospice, Konstancin-Jeziorna, Poland; Clinical Department of Oncology and Hematology, Central Clinical Hospital of the Ministry of Interior in Warsaw, Warsaw, Poland

**Keywords:** fistula, cancer, ascites, paracentesis, quality of life

## Abstract

We present the first case of peritoneal-cutaneous fistula (PCF) within ovarian carcinoma neoplastic infiltrates located inside a large abdominal hernia in a home hospice patient with massive ascites. It is an instructive case featuring a rapid diagnosis and efficient treatment of the PCF at home. As the patient refused hospitalization, she had successful paracentesis performed at home. Subsequent hydrocolloid dressing application, diuretics, and oral protein supplementation were recommended. Our intervention led to PCF closure, improved quality of life, and deepened the trust between the patient and the home hospice team. Our case demonstrates that in some instances, PCF may be efficiently treated at home, which may require paracentesis, appropriate dressings, and identification of all factors affecting healing. It also provides further support for the safety of paracentesis in home settings.

## 1 Introduction

Neoplastic peritoneal involvement is a common cause of ascites. It carries a poor prognosis and is associated with multiple complaints, significantly lowering the quality of life [1]. We describe an unusual case of peritoneal-cutaneous fistula (PCF) within the ovarian carcinoma neoplastic infiltrates located inside a large abdominal hernia in a home hospice patient with massive ascites. We successfully treated this patient at home.

## 2 Case report

**Figure 1:**
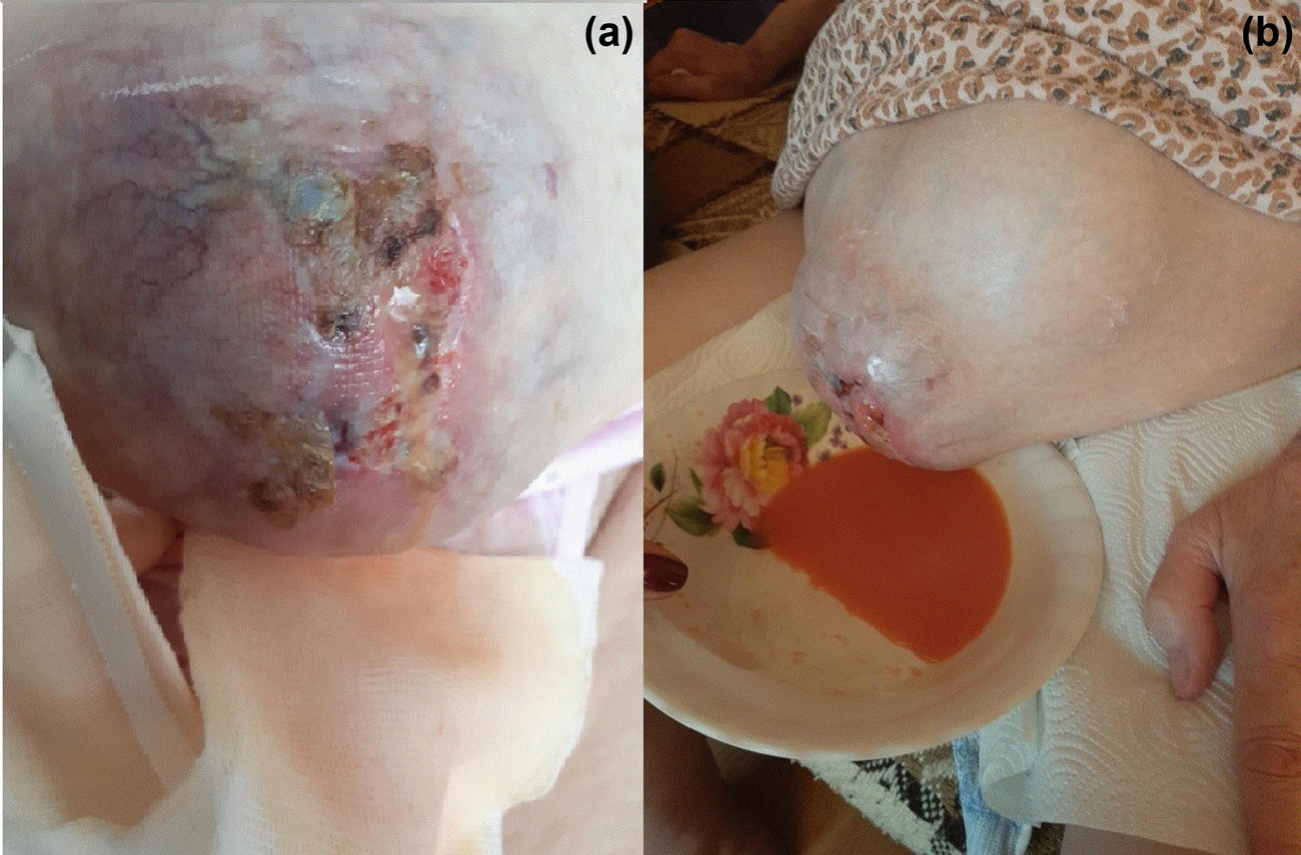
(a) Large metastatic lesion infiltrating peritoneum, omentum, fascia, subcutaneous tissue, and skin became visible due to increased ascites. (b) The cloudy orange fluid was leaking out under high pressure from the PCF.

A 77-year-old Caucasian woman with generalized ovarian cancer during chemotherapy was under home hospice care for 9 months. For most of that time, she was in a good general condition (ECOG 2). Two months before, CT had revealed dynamic progression of multiple metastatic foci in the peritoneum. Massive skin infiltration became visible in the previously diagnosed large abdominal hernia. CT scan revealed this tumor to be a part of a large metastatic lesion infiltrating the peritoneum, omentum, fascia, subcutaneous tissue, and skin (Figure 1a). Also, formerly moderate and well-controlled ascites increased. She started the fourth line of palliative chemotherapy with gemcitabine. Seven days after her last chemotherapy, she urgently called a hospice physician due to profuse leakage from the metastatic skin tumor. A few hours earlier, a slight leakage of turbid content occurred. A hospice nurse recommended a hydrocolloid dressing, but the fluid remained too abundant and the dressing burst.

We found the patient in a sitting position, with a bowl placed to collect the leaking fluid. Two thin streams of cloudy orange fluid were leaking out under high pressure from the skin lesion (Figure 1b).

The patient was nervous, dysphoric, and anxious, but in a relatively good general condition. Her vital functions were normal, and her abdomen was moderately tight with pronounced ascites but painless. Peristalsis was normal, and no peritoneal symptoms were present. We recognized a PCF based on the interview, physical examination, and the last computed tomography. Massive ascites led to an increase in intra-abdominal pressure and contributed to the fistula’s disclosure within the skin’s neoplastic infiltration. The latter developed inside the hernia and may have undergone degenerative changes in the course of local progression and chemotherapy.

**Figure 2:**
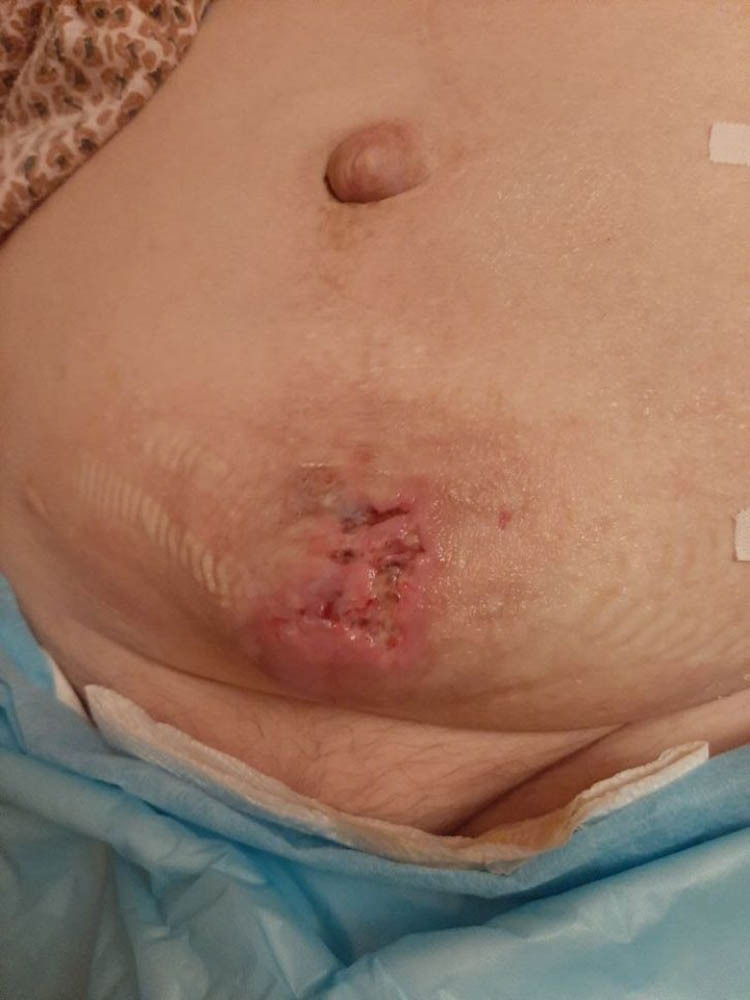
Local condition after PCF closure.


The patient refused hospitalization. During the COVID-19 pandemic, she would be separated from her family. After obtaining informed consent, and at the patient’s request, we started treatment at home. It was crucial to reduce the fluid pressure in the abdominal cavity. Under local anesthesia and following the antisepsis, we performed therapeutic paracentesis. The puncture point was determined by the percussion method and careful palpation, higher and laterally than typical, to avoid palpably perceptible masses. We evacuated 5 L of a cloudy, rusty orange exudate with the same appearance as that described above (plus 0.5 L of previous spontaneous leakage). The macroscopic appearance of the fluid could relate to decay/necrotic changes with possible blood and fibrin admixtures. However, no further laboratory tests seemed essential. The course of the procedure was uneventful. As the intra-abdominal pressure decreased, the leakage from the fistula stopped. We applied a dressing at the puncture site and a thick gauze dressing on the leakage site. We recommended increased protein supplementation and diuretics. As persisting leakage was minimal on the next day, we replaced the gauze with hydrocolloid dressing. The leakage subsided in 3 days (Figure 2).

**Informed consent:** Informed consent has been obtained from patient included in this study. 

## 3 Discussion

To date, very few cases of PCF have been reported. These include the following:

- a PCF secondary to a perforated Dalkon shield [2],
- a delayed PCF post-bilateral nephrectomy [3],
- a delayed PCF from non-retrieved gallstones during laparoscopic cholecystectomy [4],
- a PCF from spilled gall bladder calculus following laparoscopic cholecystectomy [5],
- a PCF secondary to skin excoriation from a large chronic incisional hernia [6], and
- a PCF secondary to gallstone dropped at laparoscopic cholecystectomy 20 years earlier [7].

There are no data on the incidence of PCF. We present the first description of this rare complication within vast neoplastic infiltrates of the integuments. Thus, our report is also the first concerning PCF in an oncological patient, the first within an abdominal hernia, and the first in a hospice setting. The crucial factors contributing to the occurrence of a fistula were skin weakening due to replacement of healthy tissues with neoplastic tissue, location within the sizeable abdominal hernia, and ascites. Contributing factors could also be impaired skin blood supply due to peritoneal fluid pressure, cachexia (although her albumin level slightly decreased), and everyday local micro-injuries.

Medical textbooks rarely address such issues. Surgical intervention was not an option in our patient, as peritoneal and skin infiltrates were very massive. The risk of wound dehiscence was high, and the estimated survival time short. Due to the immense danger of perforation, radiotherapy was ruled out. Similarly, our patient had chemotherapy canceled for the same reasons.

We took active and minimally invasive measures at home. Paracentesis lowered the intra-abdominal pressure, which brought the PCF walls closer and allowed its closure. Hydrocolloid dressing favored healing, while protein supplementation and diuretics supported the treatment.

We perceive our case as extremely instructive:

1. Medical staff should be aware of PCF formation in patients with massive metastases of the peritoneum and skin. It seems that abdominal hernia may represent an additional risk. Spontaneous external discharge of cloudy fluid in a patient with ascites suggests PCF. However, an abscess and entero-cutaneous fistula should always be ruled out.
2. Paracentesis, neutralizing all factors affecting healing, and appropriate dressings are crucial.
3. Paracentesis is easy but time-consuming. Data on its safety in home conditions are scanty and based on small groups of patients [8]. While it is easier to comply with anti-/asepsis in a hospital setting, the home environment poses a much lower microbiological risk.

## 4 Conclusion

Our intervention led to PCF closure, improved quality of life, and deepened trust between our patient and the hospice team. Our story also confirmed the possibility of efficient and safe paracentesis at home. We perceive paracentesis as crucial in our intervention. Still, the actual PCF prevalence and the safe use of paracentesis at home require more research.
